# Effects of amniotic membrane suspension in human corneal wound healing in vitro

**Published:** 2009-11-05

**Authors:** Jin A Choi, Hyun-Jin Jin, Samhyun Jung, Eunkyung Yang, Jun-Sub Choi, So-Hyang Chung, Choun-Ki Joo

**Affiliations:** 1 Department of Ophthalmology and Visual Science, Kangnam St. Mary's Hospital, College of Medicine, the Catholic University of Korea, Seoul, ROK; 2 Department of Tissue Engineering, Bioland, ROK

## Abstract

**Purpose:**

To investigate the biochemical mechanism of amniotic membrane (AM) suspension on corneal wound healing, particularly on epithelial proliferation and migration.

**Methods:**

Human corneal epithelial cells (HCECs) were cultured in media with different concentrations of AM suspension (5% and 30%), Dulbecco's Modified Eagle Medium: Nutrient Mixture F-12 (negative control), and serum containing Dulbecco's Modified Eagle Medium: Nutrient Mixture F-12 (positive control). In an effort to evaluate the migratory potential of AM, migration assays were conducted via the manual scraping of HCECs and immunocytochemical staining of cell adhesion molecules (E-cadherin). The relative expression of matrix metallopeptidase 9 (MMP9) and adhesion molecules (E-cadherin, fibronectin) was determined via reverse transcription-polymerase chain reaction (RT-PCR) and western blot analysis. The proliferative potential of AM was evaluated via a proliferation assay using 5-Bromo-2-deoxyuridine (BrdU) incorporation and western blot analysis for proliferating cell nuclear antigen (PCNA). In addition, enzyme-linked immunosorbent assay** (**ELISA) was used to measure the protein concentrations of mitogenic growth factors (e*pidermal growth factor [*EGF],**k*eratinocyte growth factor [*KGF], h*epatocyte growth factor [*HGF], and b*asic fibroblast growth factor [*bFGF]) in AM suspensions.

**Results:**

Migration assay rates were enhanced as AM concentrations increased, with statistically significant changes seen in 30% AM-treated and positive control cells, compared to negative control cells (p<0.05). RT-PCRs revealed that the expression of the MMP9 gene was upregulated by AM, and the expressions of E-cadherin and fibronectin genes were downregulated by AM. Western blot analysis demonstrated significantly higher MMP9 expression in AM-treated groups, versus significantly lower levels of E-cadherin and fibronectin expression in AM-treated groups. Immunocytochemistry showed large quantities of E-cadherin near the wound edges after 24 h of injury in the AM-treated groups. The proliferation assay showed that the BrdU positive cell counts/total cell counts (labeling index) were augmented by AM to a statistically significant degree (p<0.05 in the 30% AM and positive control groups). Western blot analysis showed that the expression cell cycle-associated protein, PCNA, increased gradually as a result of AM treatment. ELISA showed that our AM suspension contained 4 growth factors (HGF, EGF, KGF, and FGF). The amount of HGF was especially large, followed by that of EGF.

**Conclusions:**

These results demonstrate that the suspension form of AM maintains its beneficial effect on corneal epithelial wound healing in vitro, and that AM suspension leads to significant increases in corneal epithelial migration and proliferation with increasing AM concentrations.

## Introduction

After the use of the amniotic membrane (AM) as a graft for ocular surface reconstruction was first described by Kim and Tseng in 1995 [1], the popularity of this surgical procedure has increased. It has been well established that transplantation of AM as a temporary or permanent graft promotes epithelial wound healing and exerts profound anti-inflammatory and anti-scarring effects on the ocular surface [2,3]. Among these various functions of AM, AM-induced reepithelialization is believed to occur via a dual action mechanism, involving both a mechanical effect and a biochemical effect. Mechanically, AM provides a basement membrane-type substrate to the injured corneal epithelium [4-6]. Additionally, the hydration of the epithelium and the protection of the corneal epithelium from upper-lid irritation may play a role. The biochemical mechanism by which AM transplantation might influence ocular surface reepithelialization involves the activity of growth factors. The biochemical factors known to be involved in reepithelialization are e*pidermal growth factor (*EGF),**k*eratinocyte growth factor (*KGF), h*epatocyte growth factor (*HGF), and b*asic fibroblast growth factor (*bFGF)—derived from the AM epithelium [7]. These factors have been identified as key materials in epithelialization, particularly in cell migration and proliferation [8,9]. 

Extensive studies have previously been conducted involving AM grafts or patches [1-3,10]. However, the biochemical extracellular cues that induce reepithelialization by AM are currently poorly understood. This is because, clinically, AMs have generally been utilized as temporary or permanent grafts. The importance of the mechanical functions of AMs, rather than their biochemical functions, has been the predominant focus of previous studies. Additionally, the clinical applications of AM grafts or patches are generally limited to severe cases of ocular surface disease, because they require an invasive surgical procedure, which may cause a variety of suture-related complications [10]. Therefore, the development of a topically applicable AM suspension, the biochemical effect of which on corneal wound healing could be maintained for long periods, would be beneficial. The principal objective of this study was to define the biochemical role of AM suspensions on corneal epithelial wound healing, particularly with regard to epithelial proliferation and migration.

## Methods

### Amniotic membrane suspension preparation

In accordance with the tenets of the Declaration of Helsinki, after properly acquiring informed consent, human AM was obtained at the time of elective cesarean deliveries from the tissue bank of Seoul St.Mary’s Hospital (Seoul, ROK) and processed as described in a previous report [11]. In brief, AM was obtained from the placenta, which tested serologically negative for human immunodeficiency virus, hepatitis B and C viruses, and syphilis. The placentas were maintained in sterile plastic bags on ice during transfer, and were handled at all times using sterile technique. Under a lamellar-flow hood, the placentas were rinsed several times to remove excess blood clots, with 0.9% normal saline containing 50 mg/ml of penicillin, 50 mg/ml of streptomycin, 100 mg/ml of neomycin, and 2.5 mg/ml of amphotericin B. The AM were separated from the remaining chorion via blunt dissection using two sets of forceps while immersed in Earle’s Balanced Salt Solution (Gibco BRL Life Technologies, Gaithersburg, MD) containing the aforementioned antibiotics. Following separation from the chorion, the AM was sliced into smaller pieces using tenotomy scissors. For the extraction of proteins from AM, three methods were employed. First, a homogenizer was used under sterile conditions. After grinding the membrane, further protein extraction was conducted using a sonicator. Finally, the liquefied AM suspension was lyophilized to prevent damage to the AM suspension from the long preservation period, and to maintain the suspension’s bioactivity. The powdered AM was dissolved with Dulbecco's Modified Eagle Medium: F-12 medium (1:1; Welgene, ROK) and centrifuged for 10 min at 10,000 rpm. The supernatant was carefully separated and diluted to 10%, 15%, and 30% with Dulbecco's Modified Eagle Medium: Nutrient Mixture F-12 media. For the negative control, Dulbecco's Modified Eagle Medium: Nutrient Mixture F-12 media was used. The positive control’s culture reagents are listed below.

### Culture of HCECs and culture reagents

SV40-immortalized (transfected) human corneal epithelial cells (HCECs) were kindly provided by Dr. Kaoru Araki-Sasaki (Osaka University, Osaka, Japan). Cells were cultured in Dulbecco's Modified Eagle Medium: Nutrient Mixture F-12 medium containing 5% fetal bovine serum, 5 mg/ml insulin (Sigma, St. Louis, MO), 10 ng/ml human EGF (Sigma), 100 ng/ml cholera toxin (Biomol, Plymouth, PA), and 0.5% dimethyl sulfoxide (Sigma).

### Effect of amniotic membrane suspension on cell migration in vitro

Chambers in six-well plates were filled with 2.0 ml culture reagents containing 1×10^5^ cells and incubated for 36 h at 37 °C. Confluent cells in six-well plates were wounded by manual scraping four times with a yellow pipette tip to cross each other. Cells were washed once, and 2 ml of 0%, 5%, and 30% AM, and serum containing culture reagents, were placed in each well. Cell migration was microscopically assessed after 24 h of scraping, followed by 4% paraformaldehyde fixation and 0.2% crystal violet staining. Each group was assayed in triplicate. The extent of healing was determined by the ratio of the difference between the the wound areas at the zero hour and the wound areas remaining after 24 h.

### Cell proliferation assay

Cell proliferation was assessed via a 5-Bromo-2’-deoxy-uridine (BrdU) incorporation assay. BrdU staining was conducted using a 5-Bromo-2’-deoxy-uridine Labeling and Detection Kit I (Roche Diagnostics, IN). 5-Bromo-2-deoxyuridine (100 μg/ml) (BrdU; Sigma–Aldrich) was added to the culture wells 20 min prior to fixation with 70% ethanol in 50 mM glycine (pH 2.0). After washing with PBS three times, the wells were incubated with anti-BrdU antibody (1:100) for 30 min at 37 °C. The bound antibody was detected with anti anti-mouse Ig-fluorescein antibody (Alexa Fluor^®^ 488 goat anti-mouse IgG, 1/400; Invitrogen, Carlsbad, CA) for 30 min at room temperature. Hoechst (1 mg/ml, 1/1,000) staining was conducted for 10 min at RT. Digital images of each well were captured using a 100X objective. The labeling index (the BrdU-positive portion of the HCECs) was defined as BrdU-positive cells divided by the total count (done in triplicate) of cells stained with Hoechest.

### Reverse transcription-polymerase chain reaction (RT-PCR)

Total RNA was extracted from HCEC using TRIzol reagent (Invitrogen) in accordance with the manufacturer’s protocol. Total RNA (2 mg) was reverse-transcribed with AMV reverse transcriptase (Promega, Madison, WI), and the PCR was conducted with specific primers. The primers utilized for RT-PCR were as follows: Fibronectin-1 (forward: TCC GTG GTT GTA TCA GGA CT, reverse: GAC ATC TGG CTT GAT GGT TC, 270 bp); *E-cadheri*n (forward: AAC CTC TGT GAT GGA GGT CA, reverse: GGA TTG AAG ATC GGA GGA TT, 297 bp); and *hMMP9* (forward: CCT GGA GAC CTG AGA ACC AA, reverse: GGA CCA CAA CTC GTC ATC G, 549 bp). Glyceraldehyde-3-phosphate dehydrogenase (*GAPDH*) mRNA was employed as an endogenous reference to determine the integrity of the mRNA in each sample. The PCR experiments were normalized to the *GAPDH* expression. In order to assess the relative expression of E-cadherin, Fibronectin-1, and *MMP9*, their band densities were measured via densitometric analysis (Image Master VDS 2.0; Pharmacia Biotech Inc., San Francisco, CA).

### Western blot analysis

For western blot analysis, the HCECs were harvested in a RIPA buffer (25 mM Tris–HCl, pH 7.4, 1% Tween-20, 0.1% SDS, 0.5% sodium deoxycholate, 10% glycerol, 150 mM NaCl, 5 mM EDTA, 1 mM PMSF, 50 mM NaF, 1 mM Na3VO4, 1 μg/ml of aprotinin, leupeptin, and pepstatin). Protein concentrations were determined using a bicinchoninic acid assay protein assay kit (Pierce, Rockford, IL). The lysates were boiled for 5 min in a 1X sodium dodecyl sulfate sample buffer, loaded and separated onto 10% sodium dodecyl sulfate polyacrylamide gel electrophoresis gel, then transferred to a nitrocellulose membrane (Amersham Life Science, Cleveland, OH) using electrotransfer apparatus (Amersham). Approximately 5% skim milk in Tris Buffered Saline plus 0.1% Tween-20 was used as an antibody blocking and dilution buffer. The membrane was developed using enhanced chemiluminescence solution (Santa Cruz, Santa Cruz, CA). Pre-stained molecular weight standards were purchased from Elpis-Biotech (Korea). b-Actin was utilized as an endogenous reference to determine the integrity of the protein in each sample. The Antibodies employed in this study were obtained as follows: the proliferating cell nuclear antigen (PCNA) was from Santa Cruz. The Fibronectin-1 was from Santa Cruz. E-cadherin was purchased from Zymed Laboratories. MMP9 was from Santa Cruz. Actin was from Sigma.

### Immunocytochemistry

To evaluate cell adhesion at the wound margin, we utilized E-cadherin as an adhesion marker. For immunocytochemical detection of E-cadherin, HCECs were cultured in six-well chamber slides. Cells were wounded via manual scraping with a yellow pipette tip. The wounded cells were incubated for 24 h with 0% AM, 5% AM, and 30% AM, and serum containing Dulbecco's Modified Eagle Medium: Nutrient Mixture F-12. After the removal of the medium, cells were washed three times with PBS, and 1.0 ml of 4.0% paraformaldehyde was added to each well. The cells were fixed for 20 min at room temperature (RT) and blocked for 1 h with 2% bovine serum albumin (BSA). Mouse anti-E-cadherin antibody (1:1,000; Zymed) was applied and incubated for 1 h at RT. Slides were washed three times with PBS and incubated for 1 h at RT with goat-anti-mouth-Alexa 546 (Invitrogen). The cells were rinsed with PBS, and nuclei were counterstained with Hoechst 33342 (1:500) for 10 min at RT. The samples were then observed with a fluorescence microscope (Axiovert 200; Zeiss, Germany).

### ELISA for detection of growth factors from AM suspensions

AM suspensions were used for growth factor assay. First, total protein was measured using the BCA protein assay kit by the Bradford method in AM suspensions. The ratio of each growth factor to total protein (pg/mg) was calculated. Then, growth factor levels were measured using commercially available ELISA systems (EGF, KGF, HGF, bFGF; R&D Systems, Minneapolis, MN). 

### Statistical Analysis

Data was compared to the negative control (0% AM), and were expressed as mean±SD. Statistical analysis was conducted via an independent t-test between the negative control and the experimental group using SPSS (Version 14); the significance value was p<0.05.

## Results

### Effect of amniotic membrane suspension on cell migration in vitro

**Figure 1:**
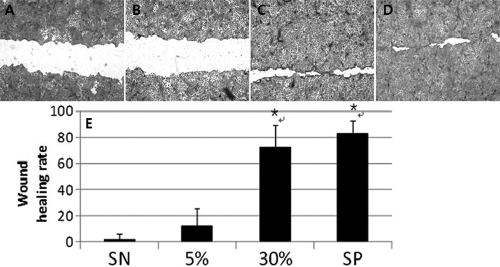
Migration assay by manual wounding of human corneal epithelial cells. HCECs were wounded by a yellow-tip in six-well plates. **A–D**: Wounds in the negative control, amniotic membrane (AM) suspensions of 5% and 30%, and the positive control (SP) healed after 24 h. **E**: Statistically significantly increased healing rates, as compared to the negative controls were noted in the 30% AM, and positive controls groups (asterisk).

In the migration assay, migration rates were increased in accordance with the AM concentration. The cornea epithelial migration rate in the negative control cells, the 5% and 30% AM-treated cells, and the positive control cells were, respectively, 2.20±0.39%, 12.37±13.60%, 73.24±16.00%, and 83.73±4.00%. The statistically significant differences in wound healing rates compared to the negative control were noted in the 30% AM-treated cells and positive control cells. (p<0.05; Figure 1).

### Gene expressions by suspension of AM

**Figure 2:**
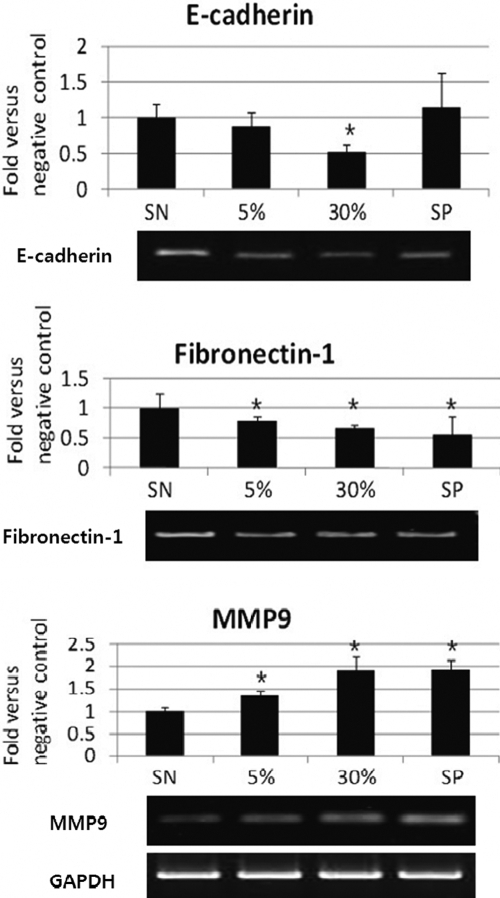
The effects of amniotic membrane suspensions on E-cadherin, Fibronectin-1, and MMP9 in corneal epithelial cells human corneal epithelial cells. RT-PCR and densitometric analyses demonstrated that human corneal epithelial cells exposed to amniotic membrane suspensions (0%, 5%, and 30%), and the positive controls (SP) evidenced increased levels of *MMP9* mRNA (p<0.05 in 5% and 30% AM, and SP). On the other hand, the expressions of E-cadherin and Fibronectin-1 were reduced in accordance with the AM concentration. The values displayed in these graphs are expressed as the means±SD of triplicate determinations in a representative experiment. PCR experiments were normalized to the levels of *GAPDH* expression.

Semiquantitative RT-PCR using normalization to GAPDH showed that the mean levels of gene expression for E-cadherin in the 5% AM, 30% AM, and positive control groups were, respectively, 0.88±0.19, 0.52±0.11, and 1.15±0.48 fold greater than that for the negative control group (Figure 2A). Statistically significant differences in gene expression, as compared to the negative control, were noted in the 30% AM group (p<0.05). Mean levels of gene expression for Fibronectin-1 in the 5%, 30%, and positive control groups were, respectively, 0.79±0.06, 0.66±0.05, and 0.55±0.31 fold greater than that for the negative control group. Statistically significant increases were noted in the 5% AM (p<0.05), 30% AM (p<0.001), and positive control groups (p<0.001; Figure 2B). Mean levels of gene expression for MMP9 in the 5% AM, 30% AM, and positive control groups were, respectively, 1.33±0.14, 1.89±0.34, and 1.92±0.24 fold greater than that for the negative control group. Statistically significant increases were noted in the 5% AM, 30% AM, and positive control groups (p<0.05; Figure 2C). Significantly more expression of MMP9 was noted in groups treated with increasing concentrations of AM, whereas significantly less expression of E-cadherin and fibronectin were observed in groups treated with AM.

**Figure 3:**
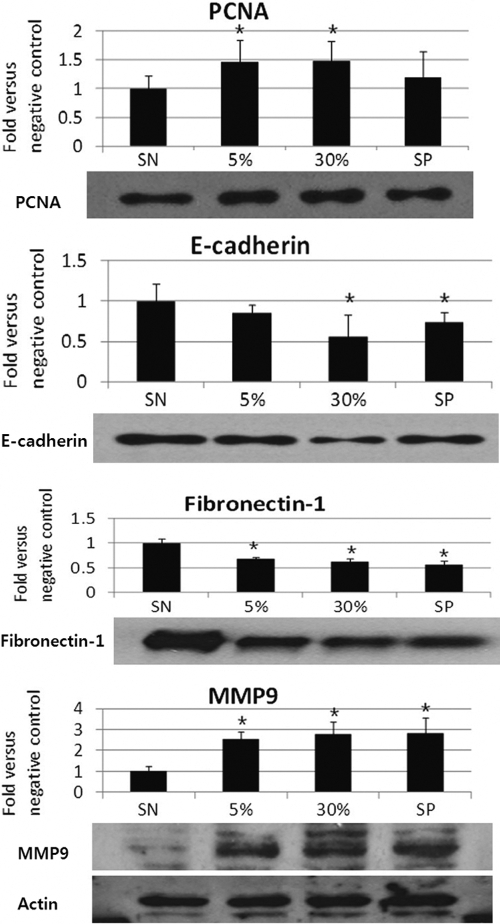
Western blot analysis of proliferating cell nuclear antigen, E-cadherin, Fibronectin-1, and MMP9 The results are representative of three independent experiments. Western blot and densitometric analyses demonstrated that HCECs exposed to AM (5% and 30%) evidenced increased levels of PCNA and MMP9 protein (p<0.05 and p<0.001, respectively), whereas HCECs exposed to AM (5% and 30%) exhibited reduced expression levels of E-cadherin and Fibronectin-1 (p<0.05 and p<0.001, respectively). The values displayed in these graphs are expressed as the means±SD of triplicate determinations in a representative experiment. Values were normalized to that of the actin expression.

The results of western blot analysis demonstrated that the expression for E-cadherin in the 5% AM, 30% AM, and positive control groups were, respectively, 0.88±0.19-, 0.52±0.11-, and 1.15±0.48 fold greater than that for the negative control group (Figure 3B). Statistically significant increases compared to negative control were noted in the 30% AM and positive control groups (p<0.05). Fibronectin-1 expression in the 5% AM, 30% AM, and positive control groups were, respectively, 0.68±0.03, 0.62±0.06, and 0.57±0.07 fold greater than that in the negative control group. Statistically significant increases were noted in the 5% AM, 30% AM, and positive control groups (p<0.001, in each group; Figure 3C). Additionally, MMP9 expression in the 5% AM, 30% AM, and positive control groups were, respectively, 2.56±0.34, 2.80±0.60, and 2.83±0.74 fold greater than that in the negative control group. Statistically significant changes were noted in the 5% AM, 30% AM, and positive control groups (p<0.001, in each group; Figure 3D). We noted a tendency toward higher MMP9 in accordance with the AM concentration, whereas lower levels of E-cadherin and fibronectin expression were noted in accordance with the AM concentration, a finding consistent with the RT-PCR results.

### Effects of amniotic membrane suspension on cellular proliferation activity

**Figure 4:**
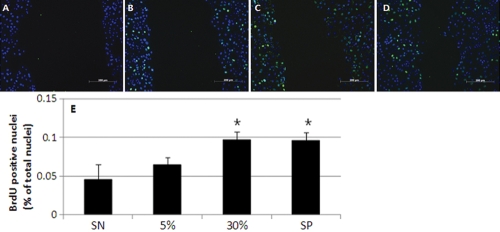
Gradual increase in proliferative potential according to the amniotic membrane concentration in human corneal epithelial cells  monolayers. 5-Bromo-2-deoxyuridine (BrdU) labeling (green) in the nucleus (blue, stained with 4',6-diamidino-2-phenylindole [DAPI]) was conducted to evaluate the proliferative potential of human corneal epithelial cells monolayers cultured at 0% amniotic membrane (**A**), 5% AM (**B**), 30% AM (**C**), and that of the positive control (**D**). **E**: Statistically significant increases in proliferative potential, as compared to the negative controls, were noted in the 30% AM and positive control groups (asterisk; N=3; Scale bar, 200 µm).

In order to determine whether the AM concentration correlated with the proliferative activity occurring in HCECs monolayers in vitro, we conducted BrdU-labeling experiments with the negative control and at different AM concentrations. The results showed that there were a number of BrdU-labeled nuclei (green) in cultured HCEC monolayers (Figure 4). The average labeling index was 4.7±3.8% in the negative control and 7.8±2.0% in the 5% AM-treated cells, 12.6±0.1% in the 30% AM-treated cells, and 15.4±1.9% in the positive control-treated cells. Statistically significant changes, compared to the negative control, were noted in the 30% AM and positive control groups (Figure 2; p=0.014 and 0.017, respectively). These results verified that the topical application of AM correlated with the proliferative potential in HCEC monolayers. Western blot analysis showed that the expression cell cycle-associated protein, PCNA, increased gradually in accordance with the concentration of AM (Figure 3A). The results of densitometric analyses demonstrated that HCECs exposed to AM exhibited increased PCNA expression, in direct relation to the concentration of AM. Significant differences (p<0.05) were noted in the 5% and 30% AM groups.

### Immunocytochemistry for E-cadherin

**Figure 5:**
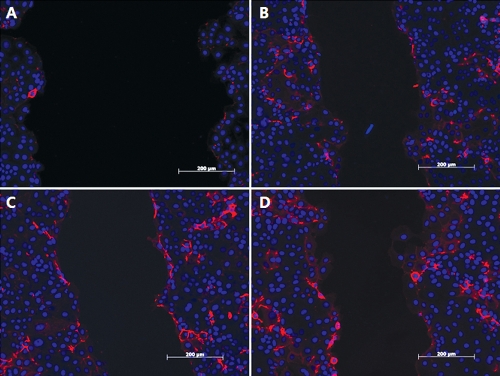
Immunocytochemical detection of E-cadherin on human corneal epithelial cells. E-cadherin expression (red) in wounded cells at different concentration of amniotic membrane suspensions (0% [**A**], 5% [**B**], 30% [**C**], and positive control groups [**D**]) are shown at 100X magnification. Cell nuclei were stained with 4',6-diamidino-2-phenylindole (blue). The levels of E-cadherin expression were increased gradually according to the amniotic membrane concentration (Scale bar, 200 μm).

Immunocytochemistry showed that large quantities of E-cadherin were deposited near the edges of the wounds after 24 h of injury (Figure 5). E-cadherin was detected near the cell boundaries and between cells. The levels of E-cadherin expression gradually increased according to the AM concentration.

### Concentrations of mitogenic growth factors in AM suspensions

Each growth factor contained in the AM suspension is shown in Table 1. HGF was most prevalent, followed by EGF. Relatively small amounts of KGF and bFGF were contained in the AM suspension.

**Table 1:** Concentrations of growth factors measured by ELISA (pg/mg).

**Growth factors**	**pg/mg**
bFGF	420.71±23.23
KGF	70.13±11.48
HGF	16430.13±174.48
EGF	2178.07±2.53

## Discussion

Renewal of the corneal epithelium is a complex process that involves the migration, proliferation, and differentiation of epithelial cells. These events perform an important role in maintaining barrier function and corneal transparency [12]. Reepithelialization, which is the regrowth of epithelia over a denuded surface, begins immediately after tissue injury [12]. It requires that epithelial cells at the edge of the wounded tissue loosen their cell–cell and cell–ECM contacts and assume a migratory phenotype [13,14]. Once the cells at the wound edge begin to migrate, the epithelial cells behind the wound edge begin to proliferate, and this continues until a new epithelium covers the damaged tissue [14,15]. Multiple MMPs have been associated with the migratory phase of wound repair, and these include MMP1, MMP3, MMP7, MMP9, MMP10, MMP14, and MMP28 [16-19]. Among these MMPs, MMP9, or gelatinase B, has also been implicated in reepithelialization after injury [12]. The epidermal growth factor (EGF) and hepatocyte growth factor (HGF) both stimulate keratinocyte migration in wound assays in vitro [16]. This cell migration is dependent on the induction of MMP9 activity, and is impaired by the presence of either the blockage of MMP9 antibodies or a general MMP inhibitor [16].

As described previously, the biochemical factors known to be involved in reepithelialization are HGF, EGF, FGF, and KGF—derived from the AM epithelium [7]. Firstly, we attempted to investigate whether our AM suspension also included these mitogenic growth factors. Our study demonstrated that the AM suspension contained these growth factors, especially a large amount of HGF, followed by EGF (Table 1). The mechanism of reepithelialization via AM suspension appears to be predominantly mediated by HGF and EGF. Second, we attempted to define the migratory mechanism of AM via the MMP9 pathway. MMP9 expression is shown to increase with increasing AM concentration in both RT-PCR and western blot analysis (Figure 2 and Figure 3). The invasion of cells into surrounding tissue is a multistep process, which additionally requires changes in cell:cell contacts and cell:substrate interactions, as well as the degradation of the extracellular matrix by metalloproteinases. The possible substrates of MMP9 include gelatin, elastin, fibronectin, collagen I/IV/V/VII/X/XI, laminin, aggrecan, vitronectin, etc. [19]. Among these substrates, we evaluated fibronectin and E-cadherin as substrates for MMP9. In our study, Fibronectin-1 and E-cadherin expression were shown to decrease in accordance with AM concentration (Figure 2 and Figure 3).

Another aspect of reepithelialization is the proliferation of epithelial cells behind the migrating wound front. In this study, western blot analysis for PCNA demonstrated increased expression according to AM concentration, where the expression of PCNA in 30% AM cells was greater than that in the positive control cells (Figure 3). The BrdU incorporation assay also indicated that proliferative potential increased in accordance with AM concentration in HCECs.

Therefore, the migratory potential of the positive control containing serum seems to be superior to that of the 30% AM suspension, whereas the proliferative potential of the positive control appears to be lower than that of the 30% AM suspension. Thus, we hypothesize that 30% AM exerts more effect on proliferation than on migration in corneal epithelial wound healing. As previously described, the process of migration requires the cells at the wound margin to loosen their cell–cell and cell–ECM contacts, as well as the degradation of the ECM molecule [13]. It is possible that the excellent proliferative ability of AM may partially disturb epithelial cell migration via the continuous production of the ECM molecule and the inhibition of ECM degradation. Immunocytochemical detection of E-cadherin showed that E-cadherin expression increases in accordance with the AM concentration, particularly on the wound margin (Figure 5). However, the total expression of E-cadherin, as determined by RT-PCR and western blot analysis (Figure 2 and Figure 3), evidences a decreasing pattern, in accordance with the AM concentration. This discrepancy may be related to the fact that wound healing is a complex process that requires the appropriate temporal and spatial expression of signaling molecules and their receptors; the expression levels of cellular adhesion molecules and ECM proteins may therefore differ.

In this study, AM suspensions were prepared via lyophilization (freeze-drying) and centrifugation in order to preserve the mitogenic growth factor proteins. Koizumi et al. [7] reported that higher levels of certain growth factors (e.g., EGF, KGF, HGF, and bFGF) were noted in the amniotic epithelium, as compared to the amniotic stroma. Thus, we did not remove the epithelium from the AM. The results of our study show that the suspension form of AM served to maintain the previously established effects of AM graft reepithelialization.

Serum drops, as well as AM transplantation techniques, represent important recent advances in the treatment of ocular surface diseases [3]. Although autologous serum also has a valuable effect on corneal wound healing, the AM has some advantages over autologous serum, as the AM has anti-inflammatory and anti-scarring properties as well. The use of autologous serum is limited, largely owing to fears of proinflammatory properties or microbial excretion [20-24]. AM has previously been shown to evidence antibacterial properties against Pseudomonas, hemolytic group A *Streptococcus*, *Staphylococcus aureus*, and *Escherichia coli* [22,25]. Gicquel et al. [26] previously reported that early amniotic membrane transplantation can be used as a safe adjuvant therapy during antibacterial treatment in cases of severe bacterial keratitis. Thus, AM suspension appears to be the drug of choice in obstinate cases of epithelial defects accompanying inflammation, particularly in cases in which infection is suspected. Additionally, it has been suggested that AM suspension may be utilized in scarless, fast epithelial healing in LASEK (laser epithelial keratomileusis) with the proven anti-scarring, anti-inflammatory effects of AM.

In summary, our observations indicate that the AM in suspension form facilitated the maintenance of the beneficial biochemical effect on corneal epithelial migration and proliferation in vitro, and the AM suspension exerted a positive effect on corneal epithelial migration and proliferation, in accordance with its concentration. Further detailed studies will be necessary to elucidate the exact mechanism as to how AM suspensions induce the migration and proliferation of epithelial cells.
